# Commentary: Cholinergic Nociceptive Mechanisms in Rat Meninges and Trigeminal Ganglia: Potential Implications for Migraine Pain

**DOI:** 10.3389/fneur.2017.00623

**Published:** 2017-11-21

**Authors:** Karl Messlinger

**Affiliations:** ^1^Institute of Physiology and Pathophysiology, Friedrich-Alexander-Universität Erlangen-Nürnberg, Erlangen, Germany

**Keywords:** headache, cholinergic receptors, dura mater, meningeal nociceptors, *in vitro* recordings

The paper by Irina Shelukhina et al. from Rashid Giniatullin’s group at the Virtanen Institute for Molecular Sciences of the University of Eastern Finland in Kuopio is about cholinergic mechanisms, which are thought to be relevant for the generation of headaches. Using preclinical models of meningeal nociception, the authors studied the effect of cholinergic agonists, acetylcholine (Ach) and carbachol, both activating nicotinic (nAch-R) and muscarinic Ach receptors (mAch-R), and of the nAch-R agonist, nicotine. The main preparation used was the hemisected rat skull with intact adhering dura mater *in vitro*, which has originally been invented in a similar form to study neuropeptide release from dural afferent fibers (1) and has later been modified to record from meningeal nerve fibers studying their activation and conduction properties (2, 3). In this preparation, using attached electrolyte-filled glass pipettes, single afferent C- and A-delta fibers can be recorded from a meningeal nerve, the spinosus nerve, which is located close to the mandibular division of the trigeminal ganglion. In addition, in the present paper histological staining, immunohistochemistry and calcium imaging were used to collect additional evidence for a role of parasympathetic neurons in meningeal nociception.

All cholinergic agonists increased spontaneous action potential firing. The activity induced by carbachol (250 µM) was reduced by the muscarinic antagonist atropine and the activity induced by nicotine (100 µM) was prevented by the nicotinic blocker d-tubocurarine, indicating that both nicotinic and muscarinic receptors are involved in the activation of meningeal afferents (Figure 1). Since it had earlier been shown that nicotine can also activate TRPA1 receptor channels (4), the TRPA1 antagonist HC-300033 was pre-applied but did not prevent the firing induced by nicotine, which excluded TRPA1 activation as mechanism underlying the nicotine effect. In addition to the electrophysiological recordings, degranulation of meningeal mast cells (MCs) was studied by histological means as a possible source of mediators that can activate meningeal afferents (2, 5). MC degranulation was induced by carbachol (50 µM) but not nicotine (100 µM) indicating a muscarinic mechanism (Figure 1). Finally, in cultured trigeminal ganglion neurons, representing their sensory terminals, nicotine as well as carbachol was demonstrated to induce intracellular Ca^2+^ transients at considerable proportions (34 and 71%, respectively). Detection of immunoreactivity for the Ach degradating enzymes, acetylcholinesterase and butyrylcholinesterase, in some dural nerve fibers supported their parasympathetic nature.

**Figure 1:**
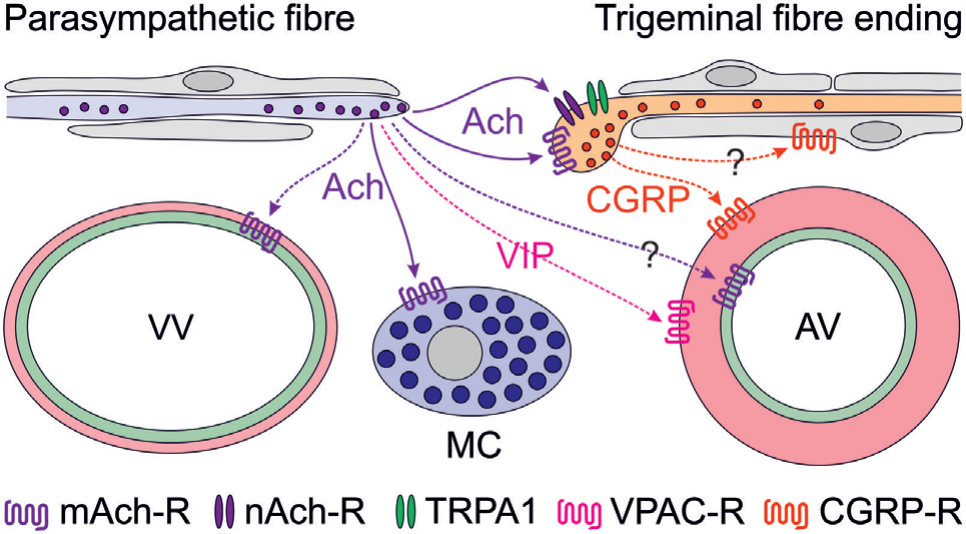
Scheme of proposed parasympathetic and trigeminal signaling in the dura mater mediated by acetylcholine (Ach) and calcitonin gene-related peptide (CGRP). Signaling by solid arrows is derived from the reviewed paper, broken arrows denote additionally discussed effects. Ach is likely to cause degranulation of mast cells (MCs) *via* muscarinic (possibly M3) Ach receptors (mAch-R) and activation of trigeminal fibers *via* mAch-R and nicotinic Ach receptors (nAch-R) but not *via* TRPA1 receptors. Activation of parasympathetic nerve fibers has previously been shown to cause plasma extravasation from venous blood vessels (VV) and to increase meningeal blood flow, most likely by dilating arterial vessels (AV). The latter may be mediated by endothelial mAch-R (possibly M3) *via* nitric oxide signaling or by vasoactive intestinal polypeptide binding to VPAC receptors on smooth arterial muscle cells, which also express CGRP receptors. Besides its prominent vasodilatory effect, CGRP may be considered to act on Schwann cells, some of which express CGRP receptors.

In earlier experiments using a rat skin *in vitro* preparation, Reeh and coworkers found that half of the identified polymodal C-fibers responded to carbachol, some also to muscarine or nicotine (6), although in a later examination muscarine turned out not to activate but rather desensitize the afferents, most likely mediated by M2 receptors (7). This was confirmed by neuropeptide release studies in isolated rat skin, where nicotine increased heat-evoked calcitonin gene-related peptide (CGRP) release but muscarine lowered basal CGRP release (8). The differing response compared to meningeal afferents may be due to different cholinergic receptor expression and to the lack of MCs in the skin responding to muscarinic agonists. It may be interesting to explore this difference regarding neurogenic inflammation in skin versus dura mater and its possible involvement in migraine mechanisms.

Cholinergic nerve fibers innervating the cranial dura mater have early been recognized (9, 10) and later functionally linked to the trigemino-parasympathetic reflex (11), which is believed to be implicated in the peripheral mechanisms of trigemino-autonomic cephalalgias (TACs) such as cluster headache (12). Experimentally, plasma extravasation in the dura mater was elicited by stimulation of the parasympathetic sphenopalatine ganglion (13) but a contribution of parasympathetic mechanisms to vasodilatation caused by direct stimulation of the dura mater in a closed cranial window could not be confirmed (14). However, there is clear clinical evidence for a contribution of parasympathetic mechanisms in some forms of TACs. Increased plasma concentrations of vasoactive intestinal polypeptide, a typical neuropeptide of parasympathetic nerve fibers, has been found during attacks of cluster headache but only occasionally in other primary headaches such as migraine (15, 16). On the other hand, facial flushing, lacrimation, and rhinorrhea, typical symptoms of parasympathetic activation, can well accompany migraine attacks (17). Thus, it is currently not entirely clear, to which extent the trigemino-parasympathetic reflex contributes to headaches other than TACs. Cortical spreading depression, which is considered to underlie the phenomena of migraine aura, has been shown to activate trigeminal afferents (18) inducing long-lasting blood flow elevation and plasma extravasation in the dura mater upon release of neuropeptides (19) (Figure 1). The blood flow increase seemed to be enhanced by the trigemino-parasympathetic pathway, which may also include vasoactive intestinal polypeptide released together with Ach from parasympathetic nerve fibers (11), though there is some discrepancy to the study mentioned above (14). The contribution of parasympathetic mediators to the direct activation of meningeal afferents is unclear so far but may be supported by the investigation reviewed here (Figure 1).

An interesting detail of the study, which has not been stressed in the paper, is the ability of CGRP to sensitize meningeal afferents to neostigmine, an inhibitor of the Ach esterase. Since CGRP receptors are present rather in peripheral Schwann cells than in sensory axons (20, 21), peripheral CGRP sensitizing mechanisms are likely indirect and may operate *via* glia–neuron signaling (Figure 1). This is an interesting topic, particularly with regard to the effects of CGRP receptor antagonists (22, 23) and CGRP-binding antibodies (24), which are believed to act peripherally outside the blood–brain barrier (25). Thus, it may be worth to look at these mechanisms using new approaches like glia–neuron cocultures, which have already been applied to study the interaction between nitric oxide and CGRP signaling (26, 27).

## Author Contributions

The author confirms being the sole contributor of this work and approved it for publication.

## Conflict of Interest Statement

The author declares that the research was conducted in the absence of any commercial or financial relationships that could be construed as a potential conflict of interest.
